# Legal Notes for Institutional Workers

**Published:** 1912-01-27

**Authors:** 


### LEGAL NOTES FOR INSTITUTIONAL WORKERS.

(The Editor will be pleased to answer Legal queries in this column.)

### MEDICAL EXAMINATION OF INJURED WORKMEN.

A curious point, which is of interest to medical practitioners who have to examine injured workmen claiming compensation, was recently considered by the House of Lords. Has the workman a right to insist that his own medical attendant shall be present during the examination? In a Scotch case it appeared that after a man had made a claim for compensation, the employers required him to submit himself for examination by their doctor. He was willing to do so provided his own medical attendant was allowed to be present. The employers refused to assent to this. Accordingly when a claim for compensation was made, the employers raised the point that the man had refused to submit to examination. This objection was upheld in the Scotch Courts, and their decision has been affirmed in the House of Lords. Lord Loreburn pointed out that it was a question of fact to be decided in each case. He (the Lord Chancellor) would hold it reasonable in nearly every case, but he could not lay it down that the workmen had an absolute right to be examined in the presence of his own doctor. Lord Shaw, who dissented, said that in view of a possible conflict of medical evidence the workman ought to be placed on the same plane. "I am sure," he added, "that 95 per cent. of medical practitioners in Scotland prefer to have the assistance of the doctor on the other side."

As illustrating the view of the medical practitioners in England, it may be mentioned that Mr. A. S. Morley, in the course of a paper read before the Medico-Legal Society, said: "In common, I believe, with most of the medical men who examine much for employers, I am always anxious that the men's doctor shall be present if it can be arranged, and wherever it is possible I give every facility for such a course. It is very unreasonable, however, for the men's solicitors to demand a fee from the employer for the presence of the medical man, who is there primarily for the man's own protection." We might add that the employer is under no obligation to pay the fee of the medical man so attending on behalf of the workman.

### THE VAGARIES OF THE CORONER'S JURY.

Service on a coroner's jury appears to be regarded by a certain class of individual as a favourable opportunity of ventilating a public grievance, or attempting to remedy a private wrong. In these days of well-nigh universal compensation, if there is a scintilla of evidence that death was caused by accident, the coroner's twelve good and lawful men will add to their verdict a rider in which they specify that A.B. met with his death while employed by C.D. These riders are doubtless prompted by the belief that they will carry due weight in a court of law. As a matter of fact the rider is not even evidence, much less is it conclusive evidence in any court of justice. This kind of rider, therefore, is comparatively harmless; but when a coroner's jury exceeds its proper function by criticising the conduct of hospital doctors who have not been before them, and have had no opportunity of delivering their defence, grave mischief may possibly be done. At the same time, it is a mistake to attach too much importance to these random verdicts. They are often unintelligible, sometimes farcical. Two classical examples are gravely recorded in the report of the Select Committee of 1893: "Death from stone in the kidney, which stone he swallowed when lying on a gravel path in a state of drunkenness"; and "Child, three months old, found dead, but no evidence whether born alive." He who reads such a verdict in his paper might be tempted to credit the following explanation of death said to have been given by an Irish jury: "Death owing to a visitation of Providence, accelerated by an injudicious post-mortem examination."

### THE DOCTOR AND HIS FEES.

It is probable that many people, instructed by ill-informed politicians, will wholly mistake the attitude of the medical profession towards one part of the Insurance Bill. It will be said, "The doctors are clamouring for more plunder. Theirs is an interested opposition." To argue with this class of person is generally futile. It is in vain to tell him that medical education is expensive; that after their hospital training the physician and surgeon has to buy experience at a heavy price—namely, years of unremunerative toil. Yet something may be done occasionally to show the most stupid person that he has no right to expect medical attendance for nothing. The hospital staff have gained their experience by attending the poor for nothing; and the private patient has to aid the cause of charity indirectly by helping the doctor to buy his experience. As example is better than precept, the following anecdote may be found useful to members of the profession who have to deal with unreasonable patients. For a working-man who came to consult him, a doctor prescribed some simple medicine which he compounded in his dispensary. He also gave certain advice and stated that his fee would be half-a-crown. The patient, who had some slight knowledge of the price of drugs, pointed out that an ounce of the active ingredient could be bought wholesale for a few pence. He accordingly protested that the fee was too high. "Very well," said the doctor, pouring the medicine down the sink, "step in and compound for yourself. I shall only charge you cost price for the drugs which you use." The patient said he could not do this, because he did not know the right proportions. The doctor was then able to explain that he had the requisite knowledge; that it had taken him years of study to obtain his qualifications; and that in charging a fee he was entitled to take his experience into account.

